# Effectiveness of Mobile Health Application-Based Interventions for Fall Prevention in Community-Dwelling Older Adults: A Systematic Review and Meta-Analysis of Randomized Controlled Trials

**DOI:** 10.3390/s26030864

**Published:** 2026-01-28

**Authors:** Saad M. Bindawas, Vishal Vennu, Maha Almarwani, Hussam M. Alsaleh, Saad M. Alsaad

**Affiliations:** 1 Department of Rehabilitation Sciences, College of Applied Medical Sciences, King Saud University, Riyadh 11433, Saudi Arabia; vvennu@ksu.edu.sa (V.V.); malmarwani@ksu.edu.sa (M.A.); 443106482@student.ksu.edu.sa (H.M.A.); 2 Department of Family & Community Medicine, College of Medicine, King Saud University, Riyadh 12372, Saudi Arabia; salsaad@ksu.edu.sa; 3 Prince Faisal Bin Bandar Chair for Geriatric Research, King Saud University, Riyadh 11451, Saudi Arabia

**Keywords:** falls prevention, mHealth app, community-dwelling older adults

## Abstract

**Highlights:**

**What are the main findings?**

- mHealth app-based interventions reduced fall risk and improved balance, strength, and fear of falling in community-dwelling older adults.
- Interventions were feasible and safe, with generally acceptable adherence and no serious adverse events.

**What are the implications of the main findings?**

- mHealth applications offer a scalable adjunct to conventional fall-prevention programs.
- Their integration into routine care may enhance access to fall prevention in community settings.

**Abstract:**

Falls are a leading cause of morbidity and loss of independence among community-dwelling older adults. Mobile health (mHealth) application (app)-based interventions have emerged as a scalable approach to fall prevention. However, evidence from individual trials remains fragmented, underscoring the need for a comprehensive quantitative synthesis. This systematic review and meta-analysis examined whether mHealth app-based interventions reduce fall incidence and improve fall-related risk factors. A systematic search of PubMed, EMBASE, CENTRAL, and Web of Science identified randomized controlled trials meeting predefined eligibility criteria. Nine trials comprising 3437 participants were included, with dual-independent data extraction, quality appraisal, and assessment of evidence certainty. Compared with usual care or control conditions, mHealth app-based interventions reduced fall risk by 11% over 12 months (risk ratio 0.89, 95% CI 0.81–0.98), corresponding to an absolute risk reduction of 6.6%. The pooled reduction in fall rate, however, did not reach statistical significance. Secondary analyses showed moderate improvements in balance, strength, and mobility, a significant decrease in fear of falling, and no serious adverse events. Overall, mHealth app-based interventions provide modest but meaningful benefits and may complement comprehensive fall-prevention strategies for older adults.

## 1. Introduction

### 1.1. Background

Falls and fall-related injuries represent persistent and leading causes of morbidity, mortality, and loss of independence among older adults worldwide [1]. With rapidly aging global populations, the clinical and economic burden of falls constitutes a major public health priority [2]. Epidemiological evidence indicates that approximately one-third of adults aged 65 years and older experience at least one fall annually. This proportion increases to nearly 50% among those aged over 80 years [2,3]. The consequences are often serious, ranging from falls requiring medical care to fractures, functional decline, long-term disability, and increased healthcare utilization [4,5,6,7]. The associated economic burden is substantial; in the United States alone, direct medical costs related to fall injuries exceed USD 50 billion annually [8,9].

Evidence from high-quality systematic reviews confirms that well-designed exercise programs are effective strategies for fall prevention, with reductions ranging from 15% to 30% [10,11,12,13]. However, translation of this evidence into population-level effectiveness has been hampered by significant implementation barriers. Conventional programs are limited by poor long-term adherence. Pooled estimates indicate that only about 21% of participants continue prescribed home exercise at 12 months [14,15]. While older adults often prefer home-based exercise, unsupervised programs lack the feedback and progression needed for optimal benefit. Although supervised in-person programs may achieve better outcomes, they face important logistical barriers. These include high costs, travel requirements, and limited accessibility, which constrain their scalability, particularly in rural and underserved settings [16,17].

The rapid expansion of mobile digital technologies, such as smartphones and tablets, offers a promising solution to these challenges. Currently, more than 65% of adults aged 65–74 years own a smartphone, and adoption continues to increase [18]. These devices provide ubiquitous access to health interventions with personalization, real-time feedback, behavioral support mechanisms, and remote supervision capabilities [19,20]. Mobile health (mHealth) interventions can deliver evidence-based exercise programs through video demonstrations, sensor-based performance monitoring, motivational prompts, and real-time feedback. These features are not available in traditional paper-based approaches [21]. Additional advantages include integration of gamification and social elements, the ability to work across geographical barriers, and significantly reduced delivery costs compared to in-person programs [22,23,24].

### 1.2. Research Gap and Objectives

While individual trials have explored the efficacy of various mHealth fall-prevention programs, a comprehensive quantitative synthesis is necessary to establish overall effectiveness and inform clinical guidelines. This systematic review and meta-analysis consolidates evidence from recent randomized controlled trials (RCTs) addressing the following questions:

(i.) Do mHealth application (app)-based interventions reduce fall incidence (both rate and risk) in community-dwelling older adults?
(ii.) Do these interventions improve key secondary outcomes, including balance, strength, and falls efficacy?
(iii.) What are the effects on adherence, feasibility, and safety?

The remainder of this paper is structured as follows: Section 2 describes the research methodology; Section 3 presents the qualitative and quantitative results; Section 4 discusses the findings; and Section 5 concludes the paper and outlines directions for future research.

## 2. Materials and Methods

This systematic review and meta-analysis of RCTs follows the Preferred Reporting Items for Systematic Reviews and Meta-Analyses (PRISMA) 2020 guidelines [25]. The protocol was registered with PROSPERO [26], and the review complies with PRISMA standards (Supplementary Table S1) for intervention reviews and adheres to the author guidelines.

### 2.1. Information Sources

Two authors (S.M.B. and V.V.) independently conducted systematic searches of PubMed, EMBASE, CENTRAL, and Web of Science to identify the relevant literature, using a combination of keywords related to falls, mobile health interventions, and older adults. Searches were completed on 15 November 2025, with no restrictions on publication date. Database-specific record counts were as follows: PubMed (*n* = 312), EMBASE (*n* = 246), CENTRAL (*n* = 137), and Web of Science (*n* = 128), totaling 823 records. Full search strategies for each database are provided in Supplementary Table S2. These procedures were conducted in accordance with PRISMA 2020 Item 7, ensuring transparency and reproducibility of the information sources.

### 2.2. Study Selection Process

Study selection involved exporting citations from an electronic database to Covidence for deduplication and hand-searching reference lists for additional trials [27]. Two independent reviewers conducted the selection in two stages, with high agreement (Cohen’s kappa > 0.75). Initially, authors S.M.B. and V.V. reviewed titles and abstracts and met to resolve disagreements. In the second phase, they separately assessed full texts and together finalized the selections, with no disagreements requiring supervisor intervention.

### 2.3. Eligibility Criteria

#### 2.3.1. Design

RCTs, including pilot and feasibility RCTs, were published in English in peer-reviewed journals.

#### 2.3.2. Population

Participants were community-dwelling older adults aged ≥60 or ≥65 years (study-specified), including those identified as high risk for falls, with a recent fall history, or with specific conditions (e.g., Parkinson’s disease) predisposing them to falls.

#### 2.3.3. Interventions

Interventions involved mobile digital technology (smartphones or tablets) with content focused on exercise, balance training, functional rehabilitation, or fall-prevention education.

#### 2.3.4. Comparator

Usual care, paper-based instructions, health education alone, or active control lacking key exercise components were included.

#### 2.3.5. Outcomes

Primary outcomes were fall incidence metrics—fall rate (Incidence Rate Ratios; IRRs) and proportion experiencing ≥ 1 fall (risk ratios; RRs). Secondary outcomes included balance measured with the Berg Balance Scale (BBS) and Timed Up and Go (TUG), lower limb strength (Chair Stand Test), falls efficacy, fear of falling, adherence, and safety.

#### 2.3.6. Time

##### No Limitations

Articles were excluded from the study if they were not RCTs, involved populations other than older adults, did not identify as high risk for falls or recent fall history, included individuals with severe cognitive impairment or those in long-term institutional care, were protocols, lacked full-text availability, or involved non-mobile app-based intervention studies. Additional exclusions applied to articles not published in English, the gray literature, unpublished works, and conference or meeting reports.

### 2.4. Methodological Quality and Risk of Bias Assessment

The Physiotherapy Evidence Database (PEDro) Scale evaluated internal validity and statistical quality on a 0–10 scale [28,29], and is reported here descriptively for comparison with the rehabilitation literature. Formal judgments of risk of bias were made using the Cochrane Risk of Bias (RoB) 2.0 tool, which assesses five domains—randomization, deviations from intended interventions, missing outcome data, outcome measurement, and selective reporting—categorized as low risk, high risk, or some concerns. Overall trial risk was determined using the RoB 2.0 algorithm [30].

### 2.5. Data Extraction

Data extraction for the included trials was conducted independently by five authors (V.V., S.M.B., M.A., H.A., and S.M.A.) using a standardized form (Supplementary Table S3). Each author handled data from 9 trials and collaborated to address disagreements. Extracted information encompassed study design, setting, follow-up duration, participant demographics, intervention specifics, comparator details, outcomes with effect estimates, adverse events, and adherence, with efforts made to contact study authors for any missing data.

### 2.6. Data Synthesis and Analysis

Data synthesis was performed using the generic inverse variance method [31] under a random-effects model (DerSimonian–Laird estimator) implemented in RevMan version 5.4 (Copenhagen, Denmark) and the metafor package in R (Auckland, New Zealand). Effect estimates were expressed as IRR for fall rates (falls per person-year), RR for dichotomous outcomes (proportion of participants experiencing ≥ 1 fall), and standardized mean differences (SMDs; Hedges’ *g*) for continuous outcomes [31,32]. Statistical heterogeneity across studies was quantified using the I^2^ statistic, with values of <25%, 25–50%, and >50% interpreted as low, moderate, and substantial heterogeneity, respectively [31,33]. The certainty of evidence for each pooled estimate was evaluated using the GRADE approach (Supplementary Table S4), considering study design, RoB, inconsistency, indirectness, imprecision, and potential publication bias [34].

Sensitivity analyses included comparisons of fixed versus random-effects models, the Hartung–Knapp adjustment for small sample sizes, restriction to trials at low RoB, and subgroup analyses stratified by intervention modality [31,35]. The number needed to treat (NNT) was derived from the absolute risk reduction using the control event rate from the Safe Step trial (59.6% fall proportion), with the baseline risk and calculation method explicitly reported (Supplementary Table S5).

## 3. Results

### 3.1. Included Studies

**Figure 1:**
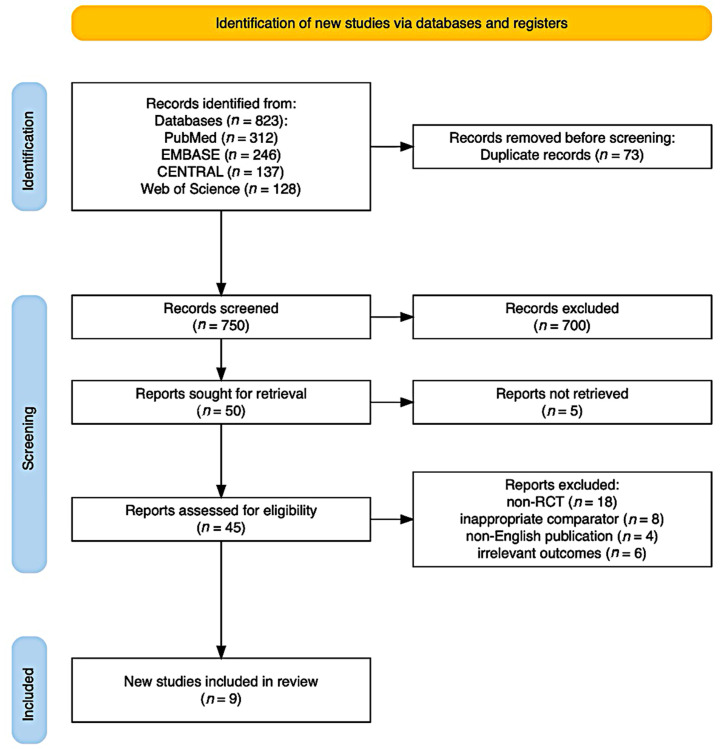
PRISMA 2020 flow diagram.

The search yielded 823 records, of which 750 were unique after deduplication. Of these, 705 were excluded. Full-text assessment of 45 articles led to the exclusion of 36 studies for reasons including non-RCT design (18), inappropriate comparator (8), non-English publication (4), and irrelevant outcomes (6). Nine RCTs [1,4,5,6,7,8,9,10,11] were included based on the criteria (Figure 1).

### 3.2. Study Characteristics

The nine included trials varied in sample size (29–1628 participants), setting, and target populations, encompassing community-dwelling older adults, post-hip-fracture patients, and individuals with Parkinson’s disease. Mean ages ranged from 64.7 to 79.5 years, and the proportion of female participants ranged from 51.7% to 100%. Interventions differed in format, frequency, and duration, including self-managed apps, telerehabilitation, gamified group programs, and social media-based education, with comparators ranging from usual care to educational materials. Follow-up periods varied from 3 weeks to 24 months, and primary outcomes included fall rates, incidence of ≥1 fall, balance, strength, mobility, fear of falling, and fall-prevention knowledge (Table 1).

### 3.3. Classification of mHealth Interventions

The included trials encompassed diverse mHealth approaches, which we categorized by delivery modality, supervision level, and core features (Table 2). Delivery modalities included self-managed apps [1,4,7], telerehabilitation or telepresence programs [5,8], gamified group-based interventions [9,10], and social media-based education [6]. Supervision ranged from fully autonomous use to remotely monitored or real-time supervised sessions, while core features included combinations of exercise content, educational modules, behavioral support, and social/gamification elements. Despite this clinical heterogeneity, pooled analysis was appropriate because all studies addressed the overarching question of fall prevention, and statistical heterogeneity was negligible (I^2^ = 0%). This classification highlights potential mechanistic differences across interventions and provides a framework for future research to explore which delivery features, supervision strategies, or behavioral components most effectively reduce falls among older adults.

### 3.4. Study Quality and Risk of Bias

RoB 2.0 assessment varied by trial. StandingTall [1] and TBHE [6] had the lowest overall risk with robust randomization, allocation concealment, blinded outcome assessment, and appropriate handling of missing data. Several trials had a high risk due to attrition and completers-only analyses. Blinding of participants and personnel was not feasible across interventions (inherent to behavioral trials). Domain-level justifications are tabulated in Table 3. PEDro scale scores ranged from 4 to 8 (median: 6.5), with StandingTall scoring highest (8/10) and Park et al. 2014 scoring lowest (4/10) [9].

GRADE certainty assessment was used for evidence certainty for primary outcomes, ranging from moderate (fall rate, downgraded for imprecision, and a small number of large trials) to high (≥1 fall risk, due to direct evidence, consistent direction, and adequate precision). Secondary outcomes were rated moderate for fear of falling and balance, strength, or mobility.

### 3.5. Primary Outcome: Fall Incidence

**Figure 2:**
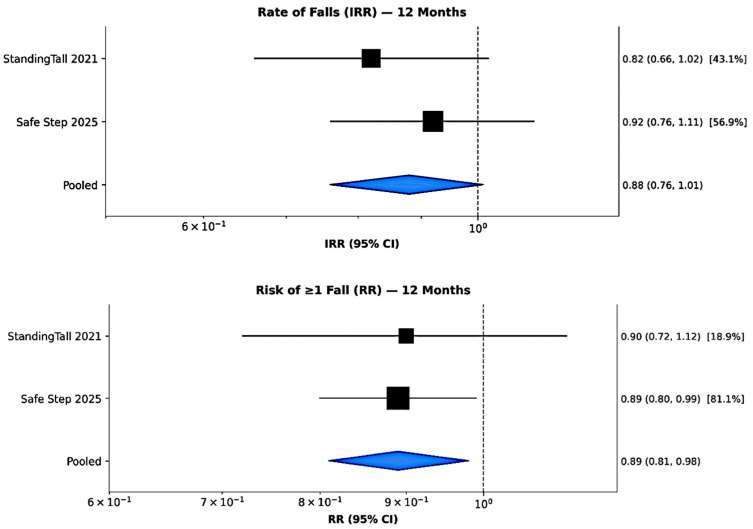
Forest plot: rate (**up**) and risk (**down**) of falls at 12 months.

The pooled analysis of two large RCTs (StandingTall and Safe Step; total *n* = 2131) demonstrated a trend toward reduced fall incidence among older adults participating in mHealth-based balance and exercise interventions. At 12 months, the pooled IRR was 0.88 (95% CI = 0.76–1.01; I^2^ = 0%; *p* = 0.070), suggesting a possible 12% reduction in fall rate that did not reach statistical significance in the intervention group than in controls (Figure 2 up). Although this reduction did not reach conventional statistical significance, both trials consistently favored the mHealth interventions. Individually, the StandingTall trial reported an IRR of 0.82 (95% CI = 0.66–1.02) [1], while the Safe Step trial found an IRR of 0.92 (95% CI = 0.76–1.11) [4]. These findings collectively suggest a meaningful, though modest, reduction in fall frequency, underscoring the potential clinical relevance of technology-based balance training even in the absence of statistical significance.

When examining the risk of experiencing at least one fall during 12 months, the pooled RR across the same trials indicated a statistically significant protective effect of mHealth interventions. The pooled RR was 0.89 (95% CI = 0.81–0.98, I^2^ = 0%, *p* = 0.020), corresponding to an 11% relative risk reduction compared with usual care (Figure 2 down). Trial-level estimates were consistent (RR = 0.90, 95% CI = 0.72–1.12) for StandingTall [1] and (RR = 0.89, 95% CI = 0.80–0.99) for Safe Step [4].

**Figure 3:**
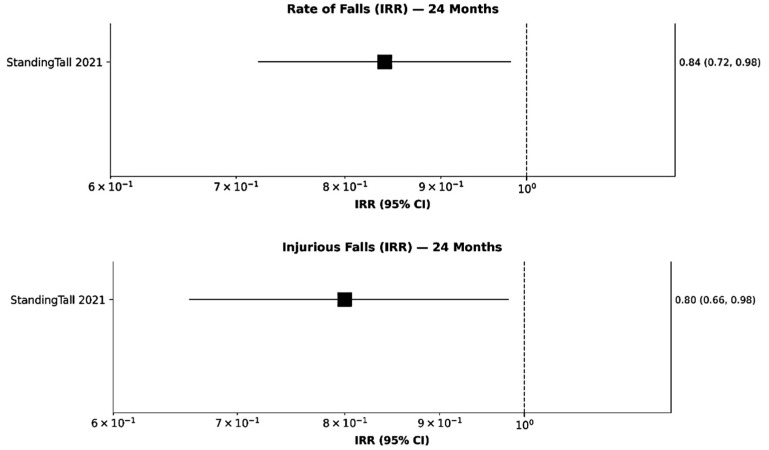
Forest plot: rate of falls (up) and injurious falls (down) at 24 months.

Importantly, longer-term follow-up data from the StandingTall trial demonstrated that the benefits of mHealth interventions may strengthen with sustained use. At 24 months, participants in the intervention group exhibited significant reductions in both overall fall rate (IRR = 0.84, 95% CI = 0.72–0.98, *p* = 0.027) (Figure 3 up) and injurious falls (IRR = 0.80, 95% CI = 0.66–0.98, *p* = 0.031) (Figure 3 down), reflecting 16% and 20% relative reductions, respectively [1]. These extended effects suggest that continued engagement with digital balance training may yield enduring improvements in stability and fall prevention. Collectively, these findings highlight the promise of mHealth approaches as scalable, sustainable, and effective tools for reducing fall risk and improving long-term safety among older adults.

### 3.6. Secondary Outcomes: Physical and Psychological Function

**Figure 4:**
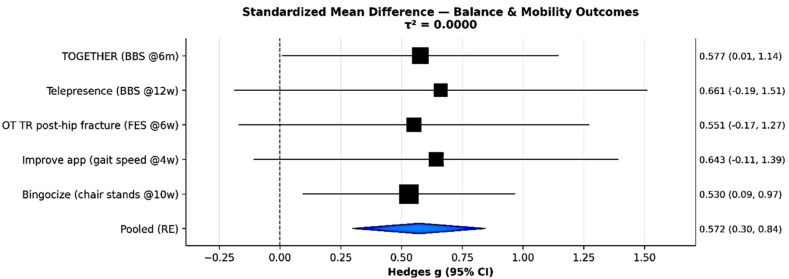
Forest plot: balance, strength, and mobility.

Across multiple RCTs, mHealth and digitally supported exercise programs demonstrated consistent and clinically meaningful improvements in lower extremity strength, balance, and mobility among older adults (Figure 4). The pooled SMD across five trials was 0.57 (95% CI = 0.30–0.84, I^2^ = 0%), representing a moderate and clinically meaningful improvement with high GRADE certainty. For example, Hong et al. (telepresence) reported a significant group-by-time interaction in BBS scores (*p* = 0.03), with intervention participants improving by 1.3 points while controls declined by 0.8 points [5]. Lugade et al. 2023 (Smartphone balance) [7] found comparable gains in gait velocity and balance between smartphone-based and traditional paper-based delivery, while Ye et al. 2022 (TBHE WeChat) [6] demonstrated significant enhancement in fall-prevention knowledge (*p* < 0.001), indicating improved home safety behaviors. Collectively, these findings affirm that mHealth interventions effectively enhance physical function and mobility in older adults, providing an accessible means of fall-prevention training.

Digital and telehealth interventions also produced favorable psychological outcomes, particularly in reducing fear of falling and enhancing self-efficacy. The pooled analysis from four trials yielded an SMD of −0.48 (95% CI = −0.75 to −0.21; I^2^ = 0%), indicating a moderate reduction in fear of falling, with *moderate* GRADE certainty after accounting for performance bias and subjective outcome measures. Individual trials reinforced these findings: Li et al. (2020) reported improved falls self-efficacy following telerehabilitation in post-hip-fracture patients [8]. Hong et al. observed significant reductions in fear of falling (*p* = 0.008) with supervised telepresence [5], and Park et al. (2014) demonstrated improved confidence and reduced fall-related anxiety through communal exercise delivered via a smart app (*p* = 0.004) [9]. Together, these results underscore the psychosocial benefits of mHealth programs, extending beyond physical gains to foster greater confidence and autonomy in older adults.

To enhance transparency and reproducibility, all arm-level data for continuous outcomes—including means, standard deviations, and sample sizes by trial and time point—are detailed in Table 4. This allows independent verification of pooled SMD calculations and facilitates alternative meta-analytic modeling. Such detailed reporting ensures methodological rigor and supports secondary analyses by researchers aiming to explore heterogeneity or conduct sensitivity testing. Overall, the convergence of physical and psychological benefits across diverse digital modalities underscores mHealth’s potential as an effective, scalable strategy for improving mobility, stability, and self-efficacy among older adults at risk of falls.

In the latter study, 53.0% of participants in the intervention group and 59.6% in the control group reported at least one fall. This corresponds to an absolute risk reduction of 6.6% and an NNT of 15 to prevent one fall over one year in populations with a baseline fall risk of approximately 60% (Table 5). This estimate is specific to populations with similar baseline risk and should not be generalized. As shown in Table 4 and Supplementary Table S5, NNT values vary across baseline fall risks ranging from 40% to 70%.

### 3.7. Adherence, Feasibility, and Safety

Adherence to mHealth fall-prevention programs varied by delivery mode but was generally favorable, with gamified and supervised formats showing the highest engagement (Table 6). Bingocize achieved exceptional attendance (93–94%) [10], while long-term self-managed programs like StandingTall (self-managed, 24 months) maintained 30–40% adherence to prescribed exercise and over 50% continued some activity—surpassing typical home-exercise retention (~21%) [1]. Similarly, Safe Step **(**self-managed**)** reported 40% exercising at least 3 days per week at 12 months [4], and supervised telepresence-based training achieved a 76.7% completion rate with minimal dropout [5]. Adherence in the TBHE WeChat program improved substantially with regular professional contact and incentives [6].

Safety outcomes across the included trials were reassuring, with intervention-related serious adverse events being exceedingly rare. In the Safe Step trial, only 0.6% of participants experienced a fall during exercise sessions, none resulting in injury [4], while StandingTall reported no serious training-related adverse events among more than 250 participants [1]. Minor, expected symptoms such as joint pain, muscle soreness, or transient dizziness occurred infrequently (1–10% across studies) and did not lead to discontinuation in any trial. Overall, these findings affirm that mHealth-based exercise interventions are safe, well-tolerated, and suitable for large-scale implementation in older adult populations.

## 4. Discussion

This systematic review and meta-analysis demonstrate that mHealth app-based interventions are effective, safe, and scalable strategies for fall prevention among community-dwelling older adults. The pooled analysis showed an 11% reduction in the proportion of participants who experienced at least one fall over 12 months. A 12% reduction in fall rate was also observed, although this did not reach statistical significance. Long-term follow-up from the StandingTall trial demonstrated sustained benefits at 24 months, including a 16% reduction in overall fall rate and a 20% reduction in injurious falls [1]. mHealth interventions additionally produced moderate, clinically meaningful improvements in balance, strength, and mobility, and significantly reduced fear of falling. Adherence rates were substantially higher (30–94%) than those reported for traditional home-based exercise programs (approximately 21%) [4]. This improvement appears driven mainly by real-time feedback, gamification, and remote supervision. Importantly, no serious adverse events were reported, supporting the safety and feasibility of digital fall-prevention strategies in older adults.

The findings of this review align with prior evidence supporting exercise-based fall prevention yet highlight the added scalability and accessibility of mHealth delivery. Traditional, supervised exercise programs reduce falls by 15–30% when implemented under controlled conditions [36,37]. The 11% relative risk reduction (RR 0.89) represents a modest but clinically meaningful benefit, corresponding to a number needed to treat (NNT) of 15. However, the non-significant 12% reduction in fall rate (IRR 0.88, *p* = 0.07) indicates that mHealth interventions are best considered adjunctive components within comprehensive, multifactorial fall-prevention programs rather than standalone solutions. Effective implementation is likely to require integration with medical optimization, environmental modifications, and periodic in-person assessment to maximize fall-risk reduction and support sustained functional independence. Digital interventions can be delivered at an estimated cost of USD 1–5 per user annually, compared to USD 50–150 for supervised group classes [38]. Consistent with recent reviews in older adult rehabilitation, mHealth-based programs achieve outcomes comparable to in-person delivery for strength, balance, and psychological well-being [39,40,41]. Studies have similarly shown that telehealth and app-based exercise programs yield equivalent or superior adherence and functional outcomes compared with traditional formats, underscoring their promise for widespread implementation in geriatric populations [42,43,44].

The nine trials included in this review exhibited substantial heterogeneity across several domains, likely reflecting the diversity of digital fall-prevention interventions for older adults. First, intervention modality varied widely, ranging from self-managed smartphone apps such as StandingTall [1] and Safe Step [4,45] to supervised telepresence programs [5], gamified group exercise sessions [9,10], and social media-based educational interventions [8]. Second, intervention duration differed markedly, from brief three-week programs to long-term interventions of up to 24 months, potentially influencing adherence, learning retention, and cumulative fall-risk reduction. Third, comparator designs varied across studies, including usual care, paper-based exercise instructions, educational videos, and active control interventions, which may have affected observed effect sizes. Finally, population characteristics ranged from general community-dwelling older adults to specific clinical populations such as individuals with Parkinson’s disease or those recovering from hip fracture, introducing further variability in baseline fall risk and responsiveness to intervention.

Despite these clinically meaningful differences, statistical heterogeneity for primary fall outcomes was low (I^2^ = 0%), suggesting that the direction and magnitude of intervention effects were generally consistent across diverse delivery formats and populations. This indicates that while the interventions differ in approach and duration, digital fall-prevention strategies may offer broadly comparable benefits in reducing fall incidence among older adults. Nevertheless, the observed heterogeneity underscores the importance of tailoring intervention selection to individual needs, functional status, and technological literacy, while also considering program duration, supervision, and engagement strategies to optimize effectiveness and adherence in real-world settings. Future research should systematically examine these moderators to refine recommendations for scalable, personalized digital fall-prevention interventions.

Preliminary evidence suggests that mHealth interventions for fall prevention are cost-effective. For example, Ambrens et al. (2022) evaluated the StandingTall program and reported an incremental cost-effectiveness ratio of AUD 20,955 per quality-adjusted life year gained, which is below commonly accepted willingness-to-pay thresholds [24]. The estimated annual per-user delivery cost of USD 1–5 compares favorably with USD 50–150 per participant for traditional supervised group exercise classes. While these findings highlight the potential economic advantage of digital fall-prevention strategies, comprehensive cost-effectiveness data across different mHealth modalities and healthcare settings remain limited, underscoring the need for further economic evaluations.

### 4.1. Clinical Implications and Implementation

From a clinical perspective, mHealth fall-prevention programs are best suited for older adults with previous falls, identified risk factors, or limited access to supervised rehabilitation [1,11]. These interventions are particularly valuable for community-dwelling individuals with basic digital literacy and access to smartphones or tablets. Programs demonstrating the greatest adherence incorporated behavioral support features (feedback, reminders, and progress tracking), social elements (group interaction and competition), gamification (points and rewards), and periodic professional contact. Integration of mHealth interventions can be conceptualized at three levels: (a) primary prevention for independent, lower-risk older adults; (b) secondary prevention or maintenance following structured physiotherapy; and (c) tertiary intervention as an adjunct to multifactorial fall risk management strategies, including medication review and environmental modification. As evidence accumulates, healthcare systems should leverage mHealth as a cost-effective, sustainable extension of conventional fall-prevention services.

Proposed Mechanism of Effect

mHealth interventions may improve balance and psychological confidence through several interrelated mechanisms. First, motor learning is facilitated by high-frequency, distributed practice with immediate feedback, which enhances skill acquisition and retention in older adults [1,45]. Second, interventions can enhance falls self-efficacy via mastery experiences, vicarious learning, and verbal persuasion, consistent with Bandura’s social cognitive theory [11]. Third, behavioral support features, such as goal-setting, progress tracking, and real-time feedback, address common adherence barriers, promoting sustained engagement [4]. Finally, social engagement in gamified or group-based formats fosters motivation and accountability, potentially amplifying intervention effects [9,10]. While these mechanisms remain largely theoretical, future trials incorporating mediation analyses could clarify how specific intervention components contribute to improvements in balance and enhanced confidence in fall prevention.

### 4.2. Strength

This review’s strengths lie in its methodological rigor and transparency. Comprehensive database searches without date restrictions, dual-independent screening and data extraction, and the use of validated quality assessment tools (RoB 2.0, PEDro) ensured robustness. Certainty of evidence was systematically rated using the GRADE framework, and both qualitative and quantitative syntheses were performed. The inclusion of definitive fall outcomes (not proxies), detailed arm-level data to enhance reproducibility, and sensitivity analyses to confirm consistency further enhances the credibility and utility of the findings. These methodological standards align with best practices in rehabilitation research [46].

### 4.3. Limitations of the Review and Evidence Base

Several limitations of the current evidence base should be noted. First, the included trials integrated diverse mHealth interventions, including self-managed apps, supervised telepresence, gamified group exercises, and social media-based educational programs, into a single analysis. To improve interpretability, we developed a classification framework (Table 2) categorizing interventions by delivery modality, supervision level, and core features (exercise, education, behavioral support, and social/gamification elements). While clinical heterogeneity exists, pooled analysis was justified because the overarching research question is consistent across trials, statistical heterogeneity was negligible (I^2^ = 0%), and effect directions were aligned, in accordance with Cochrane guidance.

Second, the evidence base remains nascent, with only two large RCTs (StandingTall, N = 503; Safe Step, N = 1628) providing definitive data on fall outcomes. Smaller trials (N < 50) offer preliminary insights but limit statistical power and preclude formal subgroup meta-analyses. Variability in intervention design, app features, supervision models, and outcome measurement introduces uncertainty regarding which specific characteristics drive effectiveness.

Third, blinding of participants and personnel was not feasible, introducing potential performance bias, particularly for subjective outcomes. Fourth, follow-up durations varied widely, limiting conclusions about long-term sustainability. Fifth, most participants were recruited from urban areas and demonstrated sufficient digital literacy, limiting generalizability to rural populations or those with limited technological experience.

Sixth, inconsistencies in fall definitions, recall intervals, and adverse event reporting across studies may affect comparability. Seventh, this review was limited to mHealth applications delivering exercise, balance, and educational interventions. It did not encompass the full spectrum of digital fall-prevention technologies, including wearable sensors, smart home systems, and AI-based prediction tools. In addition, implementation may be constrained by the digital divide, including disparities in device access, internet connectivity, digital literacy, and related equity considerations. Eighth, our restriction to English-language publications may have introduced language bias. Relevant trials published in Chinese, Japanese, Korean, or European languages could have been excluded. Future updates should consider including non-English studies with appropriate translation support.

Finally, variable follow-up durations (3 weeks to 24 months), unblinded designs, and inconsistencies in fall definitions and reporting further constrain interpretation. Collectively, these factors indicate that pooled estimates should be interpreted as representing the average effect across diverse mHealth approaches, and highlight the need for larger, well-powered trials to delineate intervention-specific mechanisms of benefit. Despite these limitations, the evidence supports mHealth interventions as a feasible, effective, and scalable strategy for fall prevention in digitally literate, community-dwelling older adults [47].

### 4.4. Recommendations for Future Research

Building on the limitations identified in this review, we propose several methodological recommendations to strengthen future mHealth fall-prevention trials. First, standardized fall definitions using the ProFaNE consensus will improve comparability across studies. Second, a minimum follow-up duration of ≥12 months (preferably 24 months) is recommended to capture sustained effects on fall incidence. Third, trials should employ core outcome sets, including fall rate, proportion of fallers, injurious falls, balance measures, and falls self-efficacy. Fourth, adherence to TIDieR reporting guidelines will enhance transparency of the intervention. Fifth, inclusive recruitment strategies are essential to ensure representation of rural and low digital literacy populations. Finally, pre-specified mediation analyses can elucidate the mechanisms underlying intervention effects, informing the design of more targeted and effective mHealth programs.

## 5. Conclusions

mHealth interventions represent a promising, scalable approach to fall prevention among community-dwelling older adults, improving balance, strength, mobility, and fear of falling. Across the included trials, there was an 11% relative risk reduction in experiencing at least one fall (RR 0.89; NNT = 15 for a 60% baseline fall risk), reflecting a modest but clinically meaningful effect. However, the pooled reduction in fall rate did not reach statistical significance (IRR 0.88, *p* = 0.07) and should be interpreted cautiously as inconclusive evidence. Accordingly, mHealth interventions are best considered adjunctive components within comprehensive, multifactorial fall-prevention programs rather than standalone solutions, ideally integrated with medical optimization, environmental modification, and periodic in-person assessment. The included trials covered diverse mHealth modalities, including self-managed apps, supervised telepresence, gamified group exercises, and social media-based education, classified by delivery modality, supervision level, and core features such as exercise, educational content, behavioral support, and social or gamification elements. While pooled analyses suggest consistent benefits, the evidence base remains limited, with only two large RCTs providing definitive fall outcomes. Small sample sizes, heterogeneous intervention designs, short follow-up periods, and participants with high digital literacy restrict generalizability, particularly to rural or technologically underserved populations. Furthermore, this review focused on exercise, balance, and educational mHealth applications. It did not cover the broader range of digital fall-prevention technologies, including wearable sensors, smart home systems, or AI-based prediction tools. Implementation may also be constrained by the digital divide, including disparities in device access, internet connectivity, and digital literacy. Future research should prioritize large, well-powered pragmatic trials with standardized outcomes, long-term follow-up, comparative evaluation of intervention components, and strategies to enhance inclusivity and accessibility. When thoughtfully implemented, mHealth interventions offer a feasible, scalable adjunct to traditional fall-prevention strategies, supporting safe and independent aging across diverse older adult populations.

## Figures and Tables

**Table 1:** Characteristics of included randomized controlled trials of mHealth fall-prevention interventions.

Study (Author, Year)	Country	Randomized, *n*	Analyzed N (ITT)	Mean Age (SD)	% Female	Primary Population	Intervention Description	Comparator	Duration	Primary Fall Outcome	Follow-Up
StandingTall (Delbaere et al., 2021) [1]	Australia	503	503	75.4 (4.2)	67%	Community-dwelling, high fall risk	Self-managed balance exercise app with video feedback	Educational videos only	24 months	Fall rate (IRR), ≥1 fall (RR), injurious falls	12 and 24 months
Safe Step (Pettersson et al., 2025) [4]	Sweden	1628	1628	75.8 (4.4)	79%	Community-dwelling age ≥70, recent fall/balance decline	Digital exercise app + monthly educational videos	Educational videos alone	12 months	Fall rate (IRR), ≥1 fall (RR)	12 months
TOGETHER (Hawley-Hague et al., 2023) [11]	UK	50	43	69.1 (8.2)	96%	Older adults referred to falls rehab services	My Activity Program app + Motivate Me (motivational messages); supervised	Usual care	6 months	Falls, balance (BBS), strength, fear falling	6 months
Hong et al. (2018) [5]	South Korea	30	23	79.5 (6.5)	100% (women)	Community-dwelling elderly women age > 65, fall risk score ≥ 14	Telepresence exercise via WebRTC (12 weeks, 3 times/week, 20–40 min)	Usual care and nutrition guidance	12 weeks	Chair stands, BBS, FOFQ, falls efficacy	12 weeks
Li et al. (2022) [8]	Hong Kong	31	30	78.8 (9.1)	93%	Post-hip-fracture, day hospital rehab, age ≥ 60	OT telerehabilitation via smartphone (Caspar Health app), 3 weeks	Paper-based home-exercise instructions	3 weeks	TUG, FR, quadriceps strength, ADL/IADL, falls efficacy	6 weeks
Ye et al. TBHE (2022) [6]	China	59	59	64.7 (6.1)	51.7%	Urban community older adults, age > 60	Teach-back health education via WeChat mini-program, 2 times/week, 8 weeks	Traditional health education via WeChat	8 weeks	Fall-prevention knowledge questionnaire	8 weeks
Lugade et al. (2023) [7]	USA	31	29	76.9 (8.6)	69%	Community-dwelling age > 65	Balance intervention via smartphone app (Improve), 3 times/week, 4 weeks	Paper-based balance exercise instructions	4 weeks	Gait velocity, balance (SOT), step characteristics	8 weeks
Park et al. (2014) [9]	South Korea	29	26	72.4 (6.3)	90%	Parkinson’s disease, non-demented, age 55–85	Communal exercise with Bingocize smart app (visual/auditory feedback), 3 times/week, 10 weeks	Usual care control	10 weeks	Gait ability (cadence, velocity), fear of falling, fall efficacy	10 weeks
Bingocize (Shake et al. 2018) [10]	USA	105	85	73.4 (7.8)	86%	Community-dwelling age > 65 from senior centers	Bingocize (exercise + health education), 2 times/week, 10 weeks	Health education only via Bingocize	10 weeks	Physical function (SPPB, chair stands, arm curls), cognition, health knowledge	10 weeks

Abbreviations: ADL—activities of daily living; BBS—Berg Balance Scale; FR—Functional Reach; FOFQ—Fear of Falling Questionnaire; IADL—instrumental activities of daily living; ITT—intention-to-treat; OT—occupational therapy; SOT—sensory organization test; TUG—Timed Up and Go; WebRTC—Web Real-Time Communication.

**Table 2:** Classification of mHealth app-based interventions for fall prevention by delivery modality, supervision, and core features.

Study (Author, Year)	Country	Randomized, *n*	Delivery Modality	Supervision Level	Core Features	Comparator	Duration	Primary Fall Outcome	Follow-Up
StandingTall (Delbaere et al., 2021) [1]	Australia	503	Self-managed app	Fully autonomous	Exercise + Behavioral support	Educational videos	24 months	Fall rate (IRR), ≥1 fall (RR), injurious falls	12 and 24 months
Safe Step (Pettersson et al., 2025) [4]	Sweden	1628	Self-managed app	Remotely monitored	Exercise + Education	Educational videos	12 months	Fall rate (IRR), ≥1 fall (RR)	12 months
TOGETHER (Hawley-Hague et al., 2023) [11]	UK	50	Self-managed app + gamification	Real-time supervised	Exercise + Behavioral support	Usual care	6 months	Falls, balance (BBS), strength, fear of falling	6 months
Hong et al. (2018) [5]	South Korea	30	Telepresence	Real-time supervised	Exercise	Usual care + nutrition guidance	12 weeks	Chair stands, BBS, FOFQ, falls efficacy	12 weeks
Li et al. (2022) [8]	Hong Kong	31	Telerehabilitation app	Remotely monitored	Exercise + Behavioral support	Paper-based home exercise	3 weeks	TUG, FR, quadriceps strength, ADL/IADL, falls efficacy	6 weeks
Ye et al. TBHE (2022) [6]	China	59	Social media platform	Fully autonomous	Education + Behavioral support	Traditional health education	8 weeks	Fall-prevention knowledge	8 weeks
Lugade et al. (2023) [7]	USA	31	Self-managed app	Fully autonomous	Exercise	Paper-based balance exercises	4 weeks	Gait velocity, balance (SOT), step characteristics	8 weeks post
Park et al. (2014) [9]	South Korea	29	Gamified group app	Real-time supervised	Exercise + Education	Usual care	10 weeks	Gait ability, fear of falling, fall efficacy	10 weeks
Bingocize (Shake et al. 2018) [10]	USA	105	Gamified group app	Real-time supervised	Exercise + Education + Social/gamification	Health education only	10 weeks	Physical function (SPPB, chair stands, arm curls), cognition, health knowledge	10 weeks

Abbreviations: ADL—activities of daily living; BBS—Berg Balance Scale; FR—Functional Reach; FOFQ—Fear of Falling Questionnaire; IADL—instrumental activities of daily living; SOT—sensory organization test; TUG—Timed Up and Go; RR—risk ratio; IRR—Incidence Rate Ratio; SPPB—Short Physical Performance Battery.

**Table 3:** Risk of bias assessment by domain (RoB 2.0).

Study (Author, Year)	Randomization	Allocation Concealment	Baseline Comparability	Deviations from Intervention	Missing Outcome Data	Outcome Measurement	Reported Results	Overall RoB	PEDro Score	Overall Quality
StandingTall (Delbaere et al., 2021) [1]	Low	Low	Low	Some concerns	Low	Low	Low	Low	8/10	Good
Safe Step (Pettersson et al., 2025) [4]	Low	Low	Low	Some concerns	Low	Low	Low	Low	7/10	Good
TOGETHER (Hawley-Hague et al., 2023) [11]	Low	Low	Low	High	Some concerns	Low	Low	Some concerns	6/10	Fair
Hong et al. (2018) [5]	Low	Low	Low	High	High	Some concerns	Low	High	5/10	Fair
Li et al. (2020) [8]	Low	Low	Low	High	Some concerns	Low	Some concerns	Some concerns	6/10	Fair
Ye et al. TBHE (2022) [6]	Low	Low	Low	High	Low	Low	Low	Low	7/10	Good
Lugade et al. (2023) [7]	Low	Low	Low	High	Low	Some concerns	Low	Some concerns	6/10	Fair
Park et al. (2014) [9]	Some concerns	Some concerns	Low	High	High	High	Low	High	4/10	Poor
Bingocize (Shake et al. 2018) [10]	Low	Low	Low	High	Some concerns	Some concerns	Low	Some concerns	6/10	Fair

Notes: All trials are at high risk for “deviations from intervention” due to the inability to blind participants/personnel (inherent to behavioral interventions). High RoB trials (Hong, Park) are primarily due to high attrition with completers-only analysis, violating the intention-to-treat principle. Low RoB trials (StandingTall, Safe Step, TBHE) demonstrated robust randomization, assessor blinding, and ITT analysis.

**Table 4:** Arm-level data for continuous outcomes: balance, strength, mobility, and psychological measures.

Study (Author, Year)	Outcome (Instrument)	Time Point	Intervention N	Intervention Mean (SD)	Control N	Control Mean (SD)	Standard Mean Difference (MSD)	Cohen’s d
StandingTall (Delbaere et al., 2021) [1]	Berg Balance Scale	Baseline	10	43.0 (6.49)	13	44.69 (3.49)	−1.69	−0.30
Safe Step (Pettersson et al., 2025) [4]	Berg Balance Scale	12 weeks	10	44.30 (6.32)	13	43.84 (3.57)	+0.46	+0.08
TOGETHER (Hawley-Hague et al., 2023) [11]	Chair Stands (reps)	Baseline	10	11.0 (4.64)	13	13.0 (2.61)	−2.0	−0.48
Hong et al. (2018) [5]	Chair Stands (reps)	12 weeks	10	19.20 (5.99)	13	14.15 (2.70)	+5.05	+1.00 *
Li et al. (2020) [8]	Berg Balance Scale	6 months	23	48.1 (4.8)	20	45.5 (5.2)	+2.6	+0.52
Ye et al. TBHE (2022) [6]	Fall-Prevention Knowledge	8 weeks	29	78.3 (8.1)	30	65.2 (9.4)	+13.1	+1.43 **
Lugade et al. (2023) [7]	Gait Velocity (m/s)	4 weeks	14	1.18 (0.21)	15	1.12 (0.18)	+0.06	+0.30
Park et al. (2014) [9]	Gait Velocity (m/min)	10 weeks	8	89.3 (11.2)	18	76.4 (13.5)	+12.9	+1.02 *
Bingocize (Shake et al. 2018) [10]	Falls Efficacy Scale	6 weeks	15	8.2 (2.1)	15	10.4 (2.8)	−2.2	−0.88 *

Notes: Positive values indicate improvement in physical/cognitive outcomes; negative values for the Falls Efficacy Scale indicate improvement (lower scores = less fear). * Large effect size (d ≥ 0.80) ** Very large effect size (d ≥ 1.20). Pooled Analysis: Standardized mean difference (SMD) across balance, strength, and mobility measures (5 trials, 8 comparisons, N ≈ 800) = 0.572 (95% CI 0.30–0.84; I^2^ = 0%; GRADE Certainty HIGH).

**Table 5:** Number needed to treat (NNT) calculation.

Parameter	Value	Source/Method
Baseline fall risk (control group)	59.6%	Safe Step trial baseline risk of experiencing ≥ 1 fall over 12 months
Intervention group fall risk	53.0%	Safe Step trial intervention group outcome
Absolute Risk Reduction (ARR)	6.6%	59.6–53.0%
Number Needed to Treat (NNT)	15	1/0.066 = 15.15
Interpretation	Treat 15 patients with the Safe Step digital app for 12 months to prevent 1 additional fall	—
95% CI for NNT	7 to 50	Derived from 95% CI of RR (0.81–0.98) and baseline risk

Important Note: NNT varies with baseline risk. In populations with a higher fall risk (e.g., 70%), NNT would be 10. In populations with lower fall risk (e.g., 40%), NNT would be 25. The NNT reported here (15) is specific to the Safe Step trial’s control group baseline risk of 59.6%. For other populations, recalculate using the formula NNT = 1/[(baseline risk) × (1 − RR)].

**Table 6:** Adherence rates and engagement metrics across trials.

Study (Author, Year)	Intervention Modality	Session Attendance/Completion	Exercise Dose Achievement	Dropout Rate	Engagement Quality	Adverse Events
StandingTall (Delbaere et al., 2021) [1]	Self-managed app (24 months)	50%+ maintained at 24 months	30–40% met 90 min/week goal by 24 months	15%	Excellent; intrinsic motivation noted	None reported
Safe Step (Pettersson et al., 2025) [4]	Self-managed app (12 months)	40% exercised ≥3 days/week @ 12 months	8.6–9.1% met prescribed dose @ 9–12 months	19.8%	Good; sustained participation	0.6% falls during exercise; no injuries
TOGETHER (Hawley-Hague et al., 2023) [11]	Supervised motivational app (6 months)	77% completed the program	High weekly reporting adherence	14%	Excellent; very satisfactory ratings	None reported
Hong et al. (2018) [5]	Telepresence (12 weeks)	76.7% completion	3 times x/week prescribed; high adherence	23%	Excellent user-friendliness	None reported
Li et al. (2020) [8]	Telerehab via smartphone (3 week)	87–90% completed	87% (intervention), 86% (control)	3%	Good; technical issues initially resolved	None reported
Ye et al. TBHE (2022) [6]	WeChat mini-program (8 weeks)	Initially poor, improved with contact	Improved with regular contact/incentives	1.67%	Good; 78.8% very/relatively satisfied	None reported
Lugade et al. (2023) [7]	Smartphone app (4 weeks)	100% completion of 12 sessions (non-dropouts)	45.0 ± 13.0 min/session	6.5%	Excellent; high enjoyment, equivalent to paper	None reported
Park et al. (2014) [9]	Communal gamified (10 weeks)	3 times/week prescribed; high adherence	Not separately quantified	10%	Excellent; social/gamified engagement	None reported
Shake et al. Bingocize (2018) [10]	Group gamified (10 weeks)	93–94% attendance across groups	Not separately quantified	19%	Excellent; sustained 10 weeks	None reported

Summary: Adherence substantially exceeded published benchmarks for older adult exercise programs (typical range 20–66%). Supervised or gamified modalities achieved the highest engagement (77–94%). Even self-managed apps showed superior adherence to traditional home exercise (~21%).

## Data Availability

The datasets presented in this article are available; requests to access them should be directed to sbindawas@ksu.edu.sa.

## Supplementary Materials

The following supporting information can be downloaded at https://www.mdpi.com/article/10.3390/s26030864/s1. Table S1: PRISMA 2020 checklist. Table S2: Complete search strategies. Table S3: Data extraction form (template). Table S4: GRADE summary of findings table. Table S5: NNT sensitivity analysis (scenario-based NNT for different baseline fall risks).

## Abbreviations

The following abbreviations are used in this manuscript:

RCTs	Randomized Controlled Trials
SMD	Standard Mean Difference
App	Application
PRISMA	Preferred Reporting Items for Systematic Reviews and Meta-Analyses
PEDro	Physiotherapy Evidence Database
IRR	Incidence Rate Ratio
RR	Relative Risk
mHealth	Mobile Health
RoB	Risk of Bias
NNT	Number Needed to Treat
BBS	Berg Balance Scale
TUG	Time Up and Go
SPPB	Short Physical Performance Battery
