# Supplementary material for: Effectiveness of Mobile Health Application-Based Interventions for Fall Prevention in Community-Dwelling Older Adults: A Systematic Review and Meta-Analysis of Randomized Controlled Trials
Source: Sensors (Basel). 2026 Jan 28;26(3):864. doi: 10.3390/s26030864 (PMC12899110; doi:10.3390/s26030864)
Supplement: Supplementary file 1 [file sensors-26-00864-s001.zip › sensors-4086856-supplementary.pdf]

Supplementary materials

Supplementary Table S1. PRISMA 2020 checklist**:** Mobile Health Application-Based Interventions for Fall Prevention in Older Adults: A Systematic Review and Meta-Analysis of Randomized Controlled Trials

Section and Topic	Item #	Checklist Item	Location Where Item Is Reported
TITLE	1	Identify the report as a systematic review and/or meta-analysis.	Title page
ABSTRACT	2	Provide a structured summary including background, objectives, data sources, study eligibility criteria, participants and interventions, study appraisal and synthesis methods, results, limitations, conclusions, and implications of key findings.	Abstract
INTRODUCTION	3	Describe the rationale for the review in the context of existing knowledge.	Introduction, paragraphs 1–4
	4	Provide an explicit statement of the objective(s) or question(s) the review addresses.	End of Introduction
METHODS	5	Indicate if a review protocol exists and where it can be accessed; if available, registration information (e.g., PROSPERO).	Methods, first paragraph
	6	Specify all study eligibility criteria (population, intervention, comparator, outcomes, study design, setting, and report characteristics).	Methods → Eligibility criteria
	7	Describe all information sources (databases, registers, websites, organizations, reference lists) and date last searched.	Methods → Information sources
	8	Present the full search strategy for at least one database, including limits used, so that it could be repeated.	Supplementary Appendix 2
	9	State the process for selecting studies (screening, eligibility, inclusion in the review, and, if applicable, inclusion in the meta-analysis).	Methods → Study selection
	10a	Describe the methods used for data collection from reports (e.g., piloted forms, independent extraction, automation tools).	Methods → Data extraction
	10b	List and define all variables for which data were sought (e.g., study characteristics, funding sources) and any assumptions made.	Methods → Data extraction
	11	Specify methods used to assess risk of bias in included studies.	Methods → Methodological quality and risk of bias assessment
	12	Specify effect measures used for each outcome (e.g., risk ratio, mean difference).	Methods → Data synthesis and analysis
	13a	Describe methods of synthesis used to combine results and rationale for their choice.	Methods → Data synthesis and analysis
	13b	Describe any methods used to assess heterogeneity (e.g., I^2, subgroup analysis).	Methods → Data synthesis and analysis
	13c	Describe any sensitivity analyses conducted to assess robustness of findings.	Methods → Data synthesis and analysis
	14	Describe any methods used to assess risk of bias due to missing results (reporting bias).	Methods → Data synthesis and analysis
	15	Describe any methods used to assess certainty (or confidence) in the body of evidence (e.g., GRADE).	Methods → Data synthesis and analysis

RESULTS	16a	Describe results of the search and selection process, ideally using a flow diagram.	Results → Study selection and characteristics; Figure 1
	16b	Cite studies that met inclusion criteria and were included in the review.	Results → Study characteristics and Tables 1–3
	17	Present characteristics of included studies (e.g., study design, participants, interventions, comparators, outcomes).	Results → Study populations, interventions, and comparators; Table 1
	18	Present assessments of risk of bias for each included study.	Results → Study quality and risk of bias; Table 2
	19	For all outcomes, present summary statistics and effect estimates, ideally using structured tables or plots.	Results → Primary and secondary outcomes; Figures 2–4
	20a	For each synthesis, summarize the characteristics and risk of bias among contributing studies.	Results → Primary outcome; Study quality section
	20b	Present results of all statistical syntheses conducted, including summary estimates and measures of heterogeneity.	Results → Primary and secondary outcomes
	20c	Present results of sensitivity analyses.	Results → Sensitivity analysis (text and appendix)
	21	Present results of assessments of reporting bias.	Methods and Results → Data synthesis (publication bias not significant)
	22	Present results of certainty assessment for each outcome (e.g., GRADE).	Results → Study quality and GRADE assessment; Appendix 4
DISCUSSION	23a	Provide a general interpretation of the results in the context of other evidence.	Discussion → Opening paragraphs
	23b	Discuss any limitations of the evidence included in the review.	Discussion → Limitations of the review and evidence base
	23c	Discuss any limitations of the review processes used.	Discussion → Limitations paragraph
	23d	Discuss implications of the results for practice, policy, and future research.	Discussion → Clinical implications and implementation; Conclusions
OTHER INFORMATION	24	Describe sources of financial or non-financial support for the review and the role of funders.	End of manuscript / Acknowledgments
	25	Declare any competing interests.	Author contributions / Declarations
	26	Report availability of data, code, and other materials used in the review.	Appendix and data availability statement

Supplementary Table S2. Complete search strategies

Database	Search String	Records Found
PubMed	("falls"[tiab] OR "accidental falls"[tiab] OR "fall prevention"[tiab] OR "fall risk"[tiab]) AND ("mHealth"[tiab] OR "m-health"[tiab] OR "mobile health"[tiab] OR "smartphone"[tiab] OR "mobile app"[tiab] OR "mobile application"[tiab] OR "telemedicine"[tiab] OR "telehealth"[tiab] OR "telecare"[tiab] OR "digital intervention"[tiab] OR "telerehabilitation"[tiab]) AND ("older adults"[tiab] OR "elderly"[tiab] OR "aged"[tiab] OR "aging"[tiab] OR "geriatric"[tiab] OR "older people"[tiab])	312
EMBASE	('falls':ti,ab OR 'accidental falls':ti,ab OR 'fall prevention':ti,ab OR 'fall risk':ti,ab) AND ('mhealth':ti,ab OR 'm-health':ti,ab OR 'mobile health':ti,ab OR 'smartphone':ti,ab OR 'mobile app':ti,ab OR 'mobile application':ti,ab OR 'telemedicine':ti,ab OR 'telehealth':ti,ab OR 'telecare':ti,ab OR 'digital intervention':ti,ab OR 'telerehabilitation':ti,ab) AND ('older adults':ti,ab OR 'elderly':ti,ab OR 'aged':ti,ab OR 'aging':ti,ab OR 'geriatric':ti,ab OR 'older people':ti,ab)	246
CENTRAL (Cochrane Library)	("falls" OR "accidental falls" OR "fall prevention" OR "fall risk") AND ("mHealth" OR "m-health" OR "mobile health" OR "smartphone" OR "mobile app" OR "mobile application" OR "telemedicine" OR "telehealth" OR "telecare" OR "digital intervention" OR "telerehabilitation") AND ("older adults" OR "elderly" OR "aged" OR "aging" OR "geriatric" OR "older people")	137
Web of Science	TS=("falls" OR "accidental falls" OR "fall prevention" OR "fall risk") AND TS=("mHealth" OR "m-health" OR "mobile health" OR "smartphone" OR "mobile app" OR "mobile application" OR "telemedicine" OR "telehealth" OR "telecare" OR "digital intervention" OR "telerehabilitation") AND TS=("older adults" OR "elderly" OR "aged" OR "aging" OR "geriatric" OR "older people")	128

Search Dates: Searches executed November 2025; no date restrictions applied.
Deduplication: Performed using Covidence automated deduplication followed by manual review.
Screening Software: Covidence (Veritas Health Innovation, Melbourne, Australia).

Supplementary Table S3. Data Extraction Form (Template)
This data extraction form is designed to capture detailed information from each included study. Please fill in the following fields as applicable.

Study design

RCT Type:	
Setting:	
Country:	
Funding Source:	

Participant characteristics

Number Randomized (N):	
Number Analyzed (N):	
Mean Age (± SD):	
Sex (Male/Female %):	
Inclusion Criteria:	
Exclusion Criteria:	
Attrition (%):	

Intervention

App Platform (e.g., iOS, Android):	
Technology Used (e.g., sensors, wearables):	
Content Type (e.g., exercise, education):	
Dose/Frequency:	
Duration (weeks/months):	
Supervision Level (e.g., self-guided, therapist-led):	
Theoretical Framework (if any):	

Comparator

Comparator Description:	
Dose/Frequency:	

Outcomes

Primary Outcomes (with effect estimates, CIs, measurement timing):	
Secondary Outcomes (with effect estimates, CIs, measurement timing):	

Adherence and safety

Adherence (e.g., % sessions completed):	
Adverse Events (if reported):	

Author contact information

Corresponding Author Name:	
Email:	
Institution:	

Notes

Additional Notes or Comments:	

Supplementary Table S4: GRADE summary of findings table

Outcome	Studies (N)	Participants	Effect (95% CI)	Certainty	Absolute Risk (per 1000)
≥1 fall at 12 months	2	2,131	RR 0.89 (0.81–0.98)	HIGH	66 fewer falls (7–183 fewer)
Fall rate at 12 months	2	2,131	IRR 0.88 (0.76–1.01)	MODERATE	—
Balance, strength, mobility	5	~800	SMD 0.572 (0.30–0.84)	HIGH	—
Fear of falling	4	~400	SMD −0.48 (−0.75 to −0.21)	MODERATE	—
Injurious falls (24 mo)	1	389	IRR 0.80 (0.66–0.98)	MODERATE	80 fewer injurious falls (4–238 fewer)

Supplementary Table S5: NNT sensitivity analysis (scenario-based NNT for different baseline fall risks)

Baseline Fall Risk	NNT
40%	25
50%	15
59.6% (Safe Step actual)	15
60%	15
70%	10

Note: Clinicians can use this table to estimate NNT for their specific population based on baseline fall risk.
